# *N*-(2-{[5-Bromo-2-(piperidin-1-yl)pyrimidin-4-yl]sulfan­yl}-4-meth­oxy­phen­yl)benzene­sulfonamide

**DOI:** 10.1107/S1600536812043668

**Published:** 2012-10-27

**Authors:** Mohan Kumar, L. Mallesha, M. A. Sridhar, Kamini Kapoor, Vivek K. Gupta, Rajni Kant

**Affiliations:** a Department of Studies in Physics, Manasagangotri, University of Mysore, Mysore 570 006, India; b PG Department of Studies in Chemistry, JSS College of Arts, Commerce and Science, Ooty Road, Mysore 570 025, India; c X-ray Crystallography Laboratory, Post-Graduate Department of Physics & Electronics, University of Jammu, Jammu Tawi 180 006, India

## Abstract

The title compound, C_22_H_23_BrN_4_O_3_S_2_, crystallizes with two mol­ecules, *A* and *B*, in the asymmetric unit. In one of these, the meth­oxy group is disordered over two sets of sites in a 0.565 (9):0.435 (9) ratio. The benzene rings bridged by the sulfonamide group are tilted relative to each other by 37.4 (1) and 56.1 (1)° in mol­ecules *A* and *B*, respectively, while the dihedral angles between the sulfur-bridged pyrimidine and benzene rings are 72.4 (1) and 70.2 (1)° for *A* and *B*, respectively. The piperidine ring adopts a chair conformation in both mol­ecules. In the crystal, inversion dimers linked by pairs of N—H⋯N hydrogen bonds occur for both *A* and *B*; the dimers are linked into [010] chains by C—H⋯O hydrogen bonds. The crystal structure also features inversion-generated aromatic π–π stacking inter­actions between the pyrimidine rings for both mol­ecules [centroid–centroid distances = 3.412 (2) (mol­ecule *A*) and 3.396 (2) Å (mol­ecule *B*)].

## Related literature

For the biological activity of sulfonamide compounds, see: Lee *et al.* (2002 ▶); Laurence (2009 ▶). For related structures, see: Rodrigues *et al.* (2011 ▶); Akkurt *et al.* (2011 ▶); Kant *et al.* (2012 ▶); Kumar *et al.* (2012 ▶).
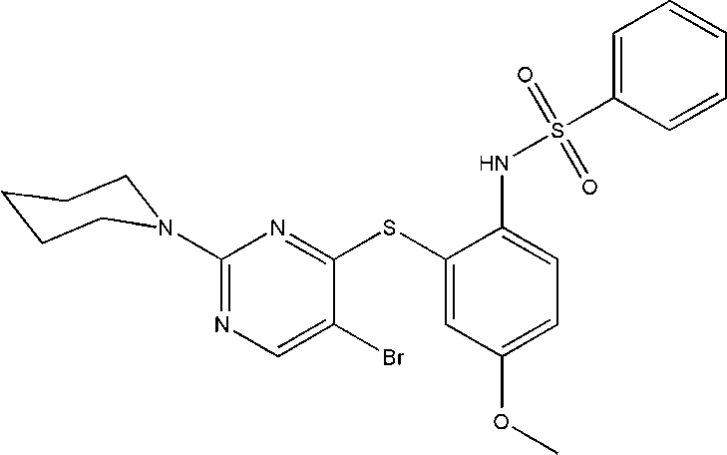


## Experimental

### 

#### Crystal data

C_22_H_23_BrN_4_O_3_S_2_
*M* *_r_* = 535.47
Triclinic, 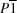

*a* = 13.6081 (6) Å
*b* = 14.5662 (5) Å
*c* = 14.7502 (7) Å
α = 74.439 (4)°
β = 69.077 (4)°
γ = 62.581 (4)°
*V* = 2405.10 (18) Å^3^
*Z* = 4
Mo *K*α radiation
μ = 1.91 mm^−1^
*T* = 293 K
0.30 × 0.20 × 0.20 mm

#### Data collection

Oxford Diffraction Xcalibur Sapphire3 CCD diffractometer
Absorption correction: multi-scan (*CrysAlis PRO*; Oxford Diffraction, 2010 ▶) *T*_min_ = 0.920, *T*_max_ = 1.000
18681 measured reflections
8017 independent reflections
5364 reflections with *I* > 2σ(*I*)
*R*_int_ = 0.032

#### Refinement

*R*[*F* ^2^ > 2σ(*F* ^2^)] = 0.042
*wR*(*F* ^2^) = 0.103
*S* = 1.02
8017 reflections
601 parameters
35 restraints
H atoms treated by a mixture of independent and constrained refinement
Δρ_max_ = 0.34 e Å^−3^
Δρ_min_ = −0.36 e Å^−3^

### 

Data collection: *CrysAlis PRO* (Oxford Diffraction, 2010 ▶); cell refinement: *CrysAlis PRO*; data reduction: *CrysAlis PRO*; program(s) used to solve structure: *SHELXS97* (Sheldrick, 2008 ▶); program(s) used to refine structure: *SHELXL97* (Sheldrick, 2008 ▶); molecular graphics: *ORTEP-3* (Farrugia, 1997 ▶); software used to prepare material for publication: *PLATON* (Spek, 2009 ▶).

## Supplementary Material

Crystal structure: contains datablock(s) I, global. DOI: 10.1107/S1600536812043668/hb6971sup1.cif

Structure factors: contains datablock(s) I. DOI: 10.1107/S1600536812043668/hb6971Isup2.hkl

Supplementary material file. DOI: 10.1107/S1600536812043668/hb6971Isup3.cml

Additional supplementary materials:  crystallographic information; 3D view; checkCIF report

## Figures and Tables

**Table 1:** Hydrogen-bond geometry (Å, °)

*D*—H⋯*A*	*D*—H	H⋯*A*	*D*⋯*A*	*D*—H⋯*A*
N8*A*—H8*A*⋯N20*A* ^i^	0.86	2.15	2.940 (4)	152
N8*B*—H8*B*⋯N20*B* ^ii^	0.78 (4)	2.28 (4)	2.974 (5)	149 (4)
C11*A*—H11*A*⋯O1*A* ^iii^	0.93	2.47	3.372 (5)	164
C10*B*—H10*B*⋯O2*B* ^iv^	0.93	2.60	3.278 (6)	131

Symmetry codes: (i) 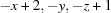; (ii) 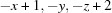; (iii) 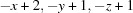; (iv) 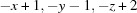.

**Fig. 1.**
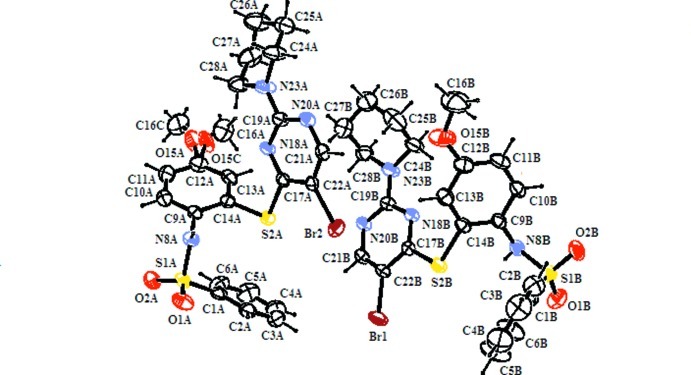
The molecular structure with displacnent ellipsoids drawn at the 40% probability level.

**Fig. 2.**
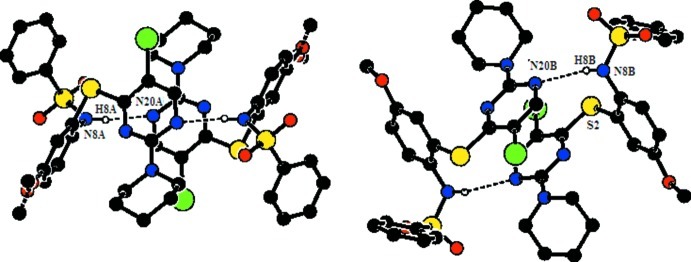
Dimer formation between the molecules.

**Fig. 3.**
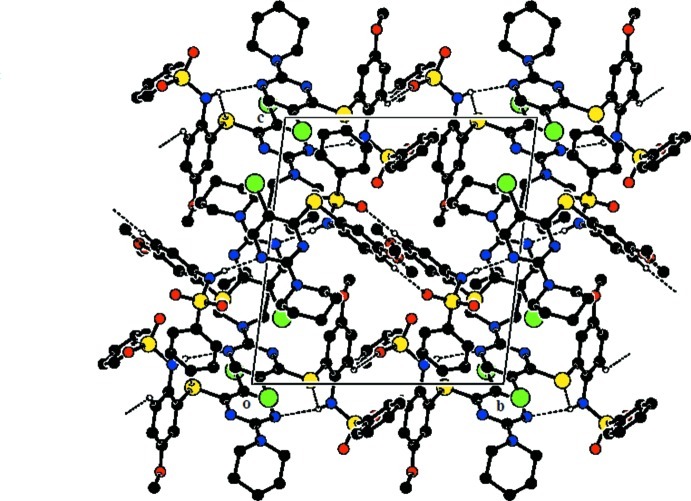
A molecular packing view of the title compound down the a axis, showing intermolecular interactions. For clarity, hydrogen atoms which are not involved in hydrogen bonding have been omitted.

## supplementary crystallographic information

### Comment

The amide and sulfonamide moieties are the constituents of many biologically significant compounds. Sulfonamide compounds are well known as antimicrobial agents and are familiar in the literature for their anti-malarial, anti-convulsant, antineoplastic, antituberculosic, antidiabetic, antiobesity. and anti-hypertensive activities [Lee *et al.*, 2002]. Sulfonamides commonly named as Sulfa drugs are the medicines capable of controlling the bacterial infections[Laurence, 2009]. In view of the importance of sulfonamide, the title compound (I) is prepared and its crystal structure is reported.

Bond lengths and angles in the title compound (Fig. 1) have normal values and are comparable with the similar crystal structures (Rodrigues *et al.*, 2011; Akkurt *et al.*, 2011; Kant *et al.*, 2012); Kumar *et al.* (2012). There are no significant differences in bond lengths and angles for molecule A & B. The molecules are twisted about the S—N bonds with (C1A—S1A—N8A—C9A) and (C1B—S1B—N8B—C9B) torsion angles of 60.5 (3) and 58.9 (4)°, respectively. The piperidine ring is in chair conformation in both the molecules. The benzene rings bridged by the sulfonamide group are tilted relative to each other by 37.4 (2) and 56.1 (1)° while the dihedral angle between the sulfur bridged pyrimidine and benzene rings is 72.4 (1) and 70.2 (1)° in molecules A and B, respectively. In one of the molecules (molecule A), methoxy group is disordered over two sets of sites in a 0.57 (1): 0.43 (1) ratio. In the crystal, molecule A is forming dimer with other molecule A by N8A—H8A ···N20A and molecule B is also forming dimer with molecule B by N8B—H8B ···N20B hydrogen bonds (Fig. 2). These dimers are further connected into chains by C10B—H10B···O2B and C11A—H11A···O1A along *a* axis (Fig. 3). In addition to these hydrogen bonds there is one intramolecular N8B—H8B···S2B hydrogen bond in molecule B (Table 1). The crystal structure also features π–π interactions between the pyrimidine rings in both the molecules, A & B [molecule A: centroid separation = 3.412 (2) Å, interplanar spacing = 3.401 Å and centroid shift = 0.27 Å; molecule B: centroid separation = 3.396 (2) Å, interplanar spacing = 3.322 Å and centroid shift = 0.70 Å].

### Experimental

The reaction of *N*-[2-(5-bromo-2-piperidin-1-yl-pyrimidin-4-yl-sulfanyl) -4-methoxy-phenyl]-benzenesulfonamide (4.87 g, 0.01 mol) and piperidine (0.86 g, 0.01 mol) were carried out in the presence of triethylamine and the reaction mixture was allowed to stir at room temperature for 6–7 h in dry dichloromethane. The progress of the reaction was monitored by TLC. Upon completion, the solvent was removed under reduced pressure and residue was extracted with ethyl acetate. The compound was purified by successive recrystallization from methanol (yield 79%, m.p. 471–473 K) to yield colourless blocks.

### Refinement

All H atoms were positioned geometrically and were treated as riding on their parent C/N atoms, with C—H distances of 0.93–0.97 Å and N—H distance of 0.86 with *U*_iso_(H) = 1.2*U*_eq_(C) or 1.5*U*_eq_(methyl C).

### Crystal data

**Table:**

C_22_H_23_BrN_4_O_3_S_2_	*Z* = 4
*M**_r_* = 535.47	*F*(000) = 1096
Triclinic, *P*1	*D*_x_ = 1.479 Mg m^−^^3^
Hall symbol: -P 1	Mo *K*α radiation, λ = 0.71073 Å
*a* = 13.6081 (6) Å	Cell parameters from 6323 reflections
*b* = 14.5662 (5) Å	θ = 3.4–29.0°
*c* = 14.7502 (7) Å	µ = 1.91 mm^−^^1^
α = 74.439 (4)°	*T* = 293 K
β = 69.077 (4)°	Block, colourless
γ = 62.581 (4)°	0.30 × 0.20 × 0.20 mm
*V* = 2405.10 (18) Å^3^	

### Data collection

**Table:**

Oxford Diffraction Xcalibur Sapphire3 CCD diffractometer	8017 independent reflections
Radiation source: fine-focus sealed tube	5364 reflections with *I* > 2σ(*I*)
Graphite monochromator	*R*_int_ = 0.032
Detector resolution: 16.1049 pixels mm^-1^	θ_max_ = 25.0°, θ_min_ = 3.4°
ω scan	*h* = −16→15
Absorption correction: multi-scan (*CrysAlis PRO*; Oxford Diffraction, 2010)	*k* = −17→17
*T*_min_ = 0.920, *T*_max_ = 1.000	*l* = −17→17
18681 measured reflections	

### Refinement

**Table:**

Refinement on *F*^2^	Primary atom site location: structure-invariant direct methods
Least-squares matrix: full	Secondary atom site location: difference Fourier map
*R*[*F*^2^ > 2σ(*F*^2^)] = 0.042	Hydrogen site location: inferred from neighbouring sites
*wR*(*F*^2^) = 0.103	H atoms treated by a mixture of independent and constrained refinement
*S* = 1.02	*w* = 1/[σ^2^(*F*_o_^2^) + (0.0369*P*)^2^ + 0.8571*P*] where *P* = (*F*_o_^2^ + 2*F*_c_^2^)/3
8017 reflections	(Δ/σ)_max_ = 0.001
601 parameters	Δρ_max_ = 0.34 e Å^−^^3^
35 restraints	Δρ_min_ = −0.36 e Å^−^^3^

### Special details

**Table:**

Experimental. *CrysAlis PRO*, Oxford Diffraction Ltd., Version 1.171.34.40 (release 27–08-2010 CrysAlis171. NET) (compiled Aug 27 2010,11:50:40) Empirical absorption correction using spherical harmonics, implemented in SCALE3 ABSPACK scaling algorithm.
Geometry. All e.s.d.'s (except the e.s.d. in the dihedral angle between two l.s. planes) are estimated using the full covariance matrix. The cell e.s.d.'s are taken into account individually in the estimation of e.s.d.'s in distances, angles and torsion angles; correlations between e.s.d.'s in cell parameters are only used when they are defined by crystal symmetry. An approximate (isotropic) treatment of cell e.s.d.'s is used for estimating e.s.d.'s involving l.s. planes.
Refinement. Refinement of *F*^2^ against ALL reflections. The weighted *R*-factor *wR* and goodness of fit *S* are based on *F*^2^, conventional *R*-factors *R* are based on *F*, with *F* set to zero for negative *F*^2^. The threshold expression of *F*^2^ > σ(*F*^2^) is used only for calculating *R*-factors(gt) *etc*. and is not relevant to the choice of reflections for refinement. *R*-factors based on *F*^2^ are statistically about twice as large as those based on *F*, and *R*- factors based on ALL data will be even larger.

### Fractional atomic coordinates and isotropic or equivalent isotropic displacement parameters (Å^2^)

**Table:**

	*x*	*y*	*z*	*U*_iso_*/*U*_eq_	Occ. (<1)
C1A	0.9685 (3)	0.2480 (3)	0.7938 (3)	0.0560 (10)	
C2A	0.8683 (5)	0.3377 (3)	0.8080 (3)	0.0820 (14)	
H2A	0.8638	0.3983	0.7649	0.098*	
C3A	0.7749 (5)	0.3370 (4)	0.8862 (4)	0.0990 (17)	
H3A	0.7071	0.3970	0.8950	0.119*	
C4A	0.7816 (5)	0.2491 (5)	0.9504 (4)	0.0934 (16)	
H4A	0.7185	0.2491	1.0031	0.112*	
C5A	0.8809 (5)	0.1609 (4)	0.9373 (3)	0.0880 (15)	
H5A	0.8856	0.1010	0.9816	0.106*	
C6A	0.9744 (4)	0.1602 (3)	0.8585 (3)	0.0675 (11)	
H6A	1.0416	0.0997	0.8496	0.081*	
C9A	0.9556 (3)	0.2902 (3)	0.5727 (3)	0.0503 (9)	
C10A	0.9569 (4)	0.3794 (3)	0.5091 (3)	0.0669 (11)	
H10A	1.0235	0.3915	0.4869	0.080*	
C11A	0.8627 (4)	0.4495 (3)	0.4785 (3)	0.0740 (12)	
H11A	0.8656	0.5081	0.4347	0.089*	
C12A	0.7634 (4)	0.4334 (3)	0.5125 (3)	0.0687 (12)	
O15A	0.6783 (14)	0.5178 (12)	0.4733 (16)	0.104 (3)	0.565 (9)
C16A	0.5841 (8)	0.5017 (8)	0.5000 (8)	0.110 (4)	0.565 (9)
H16D	0.5264	0.5601	0.4733	0.166*	0.565 (9)
H16E	0.5567	0.4937	0.5701	0.166*	0.565 (9)
H16F	0.6002	0.4396	0.4763	0.166*	0.565 (9)
O15C	0.6630 (17)	0.4908 (15)	0.4905 (19)	0.091 (4)	0.435 (9)
C16C	0.6574 (12)	0.5800 (10)	0.4197 (10)	0.121 (5)	0.435 (9)
H16G	0.5825	0.6140	0.4086	0.182*	0.435 (9)
H16H	0.7147	0.5589	0.3596	0.182*	0.435 (9)
H16I	0.6711	0.6272	0.4432	0.182*	0.435 (9)
C13A	0.7588 (3)	0.3452 (3)	0.5764 (3)	0.0617 (10)	
H13A	0.6910	0.3350	0.5999	0.074*	
C14A	0.8561 (3)	0.2725 (3)	0.6048 (2)	0.0516 (9)	
C17A	0.8532 (3)	0.0872 (3)	0.6017 (2)	0.0447 (8)	
C19A	0.8363 (3)	0.0788 (3)	0.4561 (3)	0.0523 (9)	
C21A	0.8764 (3)	−0.0689 (3)	0.5619 (3)	0.0517 (9)	
H21A	0.8922	−0.1396	0.5781	0.062*	
C22A	0.8721 (3)	−0.0174 (3)	0.6297 (2)	0.0450 (8)	
C24A	0.8154 (4)	0.0798 (3)	0.2969 (3)	0.0885 (15)	
H24C	0.8793	0.0806	0.2403	0.106*	
H24D	0.8288	0.0075	0.3222	0.106*	
C25A	0.7108 (5)	0.1291 (4)	0.2668 (4)	0.0983 (16)	
H25A	0.6497	0.1160	0.3198	0.118*	
H25B	0.7220	0.0983	0.2113	0.118*	
C26A	0.6750 (5)	0.2438 (4)	0.2394 (4)	0.117 (2)	
H26C	0.6004	0.2745	0.2265	0.140*	
H26D	0.7300	0.2575	0.1801	0.140*	
C27A	0.6684 (5)	0.2930 (4)	0.3207 (4)	0.1037 (18)	
H27A	0.6500	0.3667	0.3000	0.124*	
H27B	0.6074	0.2860	0.3778	0.124*	
C28A	0.7793 (5)	0.2426 (3)	0.3468 (3)	0.0872 (15)	
H28C	0.8387	0.2564	0.2922	0.105*	
H28D	0.7716	0.2715	0.4023	0.105*	
N8A	1.0569 (3)	0.2203 (2)	0.6031 (2)	0.0560 (8)	
H8A	1.1033	0.1651	0.5752	0.067*	
N18A	0.8354 (2)	0.1346 (2)	0.5161 (2)	0.0494 (7)	
N20A	0.8594 (2)	−0.0238 (2)	0.4749 (2)	0.0527 (8)	
N23A	0.8120 (3)	0.1302 (2)	0.3711 (3)	0.0772 (11)	
O1A	1.0816 (3)	0.3475 (2)	0.6652 (2)	0.0901 (10)	
O2A	1.1862 (2)	0.1570 (2)	0.7068 (2)	0.0866 (9)	
S1A	1.08444 (10)	0.24479 (9)	0.69014 (8)	0.0652 (3)	
S2A	0.85282 (9)	0.15692 (8)	0.68323 (7)	0.0583 (3)	
Br2	0.89499 (4)	−0.08786 (3)	0.75225 (3)	0.07432 (16)	
C1B	0.7248 (4)	−0.4807 (3)	1.1382 (3)	0.0621 (11)	
C2B	0.8015 (5)	−0.5570 (4)	1.0799 (3)	0.0874 (14)	
H2B	0.7761	−0.5815	1.0444	0.105*	
C3B	0.9179 (5)	−0.5978 (4)	1.0742 (4)	0.1111 (19)	
H3B	0.9707	−0.6505	1.0355	0.133*	
C4B	0.9542 (5)	−0.5608 (6)	1.1251 (5)	0.124 (2)	
H4B	1.0322	−0.5886	1.1212	0.149*	
C5B	0.8804 (6)	−0.4850 (5)	1.1809 (5)	0.135 (2)	
H5B	0.9067	−0.4599	1.2151	0.162*	
C6B	0.7642 (4)	−0.4440 (4)	1.1872 (5)	0.112 (2)	
H6B	0.7125	−0.3908	1.2256	0.134*	
C9B	0.6003 (3)	−0.3267 (2)	0.9702 (3)	0.0482 (9)	
C10B	0.5698 (3)	−0.3699 (3)	0.9165 (3)	0.0599 (10)	
H10B	0.5161	−0.3989	0.9479	0.072*	
C11B	0.6192 (4)	−0.3700 (3)	0.8163 (3)	0.0683 (12)	
H11B	0.5992	−0.3999	0.7810	0.082*	
C12B	0.6971 (4)	−0.3264 (3)	0.7694 (3)	0.0684 (11)	
C13B	0.7305 (3)	−0.2857 (3)	0.8216 (3)	0.0616 (10)	
H13B	0.7854	−0.2581	0.7897	0.074*	
C14B	0.6820 (3)	−0.2860 (2)	0.9222 (3)	0.0494 (9)	
C16B	0.7037 (5)	−0.3354 (6)	0.6099 (4)	0.145 (3)	
H16A	0.7162	−0.4075	0.6208	0.217*	
H16B	0.7405	−0.3178	0.5430	0.217*	
H16C	0.6226	−0.2928	0.6237	0.217*	
C17B	0.6646 (3)	−0.0985 (3)	0.9436 (2)	0.0434 (8)	
C19B	0.5571 (3)	0.0341 (3)	0.8500 (3)	0.0504 (9)	
C21B	0.6364 (3)	0.0740 (3)	0.9370 (3)	0.0531 (9)	
H21B	0.6486	0.1227	0.9565	0.064*	
C22B	0.6874 (3)	−0.0282 (3)	0.9720 (2)	0.0464 (9)	
C24B	0.4822 (4)	−0.0133 (3)	0.7486 (3)	0.0765 (13)	
H24A	0.4020	0.0083	0.7526	0.092*	
H24B	0.5074	−0.0803	0.7881	0.092*	
C25B	0.5520 (4)	−0.0234 (5)	0.6457 (5)	0.114 (2)	
H25C	0.5402	−0.0716	0.6207	0.137*	
H25D	0.6329	−0.0516	0.6427	0.137*	
C26B	0.5200 (6)	0.0801 (6)	0.5830 (4)	0.136 (3)	
H26A	0.4421	0.1042	0.5789	0.164*	
H26B	0.5714	0.0726	0.5174	0.164*	
C27B	0.5279 (5)	0.1599 (5)	0.6262 (4)	0.1155 (19)	
H27C	0.6078	0.1408	0.6212	0.139*	
H27D	0.4998	0.2279	0.5887	0.139*	
C28B	0.4593 (4)	0.1662 (3)	0.7312 (3)	0.0725 (12)	
H28A	0.4717	0.2125	0.7583	0.087*	
H28B	0.3780	0.1943	0.7358	0.087*	
N8B	0.5458 (3)	−0.3251 (3)	1.0724 (3)	0.0559 (9)	
N18B	0.5992 (2)	−0.0683 (2)	0.8847 (2)	0.0486 (7)	
N20B	0.5702 (2)	0.1085 (2)	0.8768 (2)	0.0510 (7)	
N23B	0.4937 (3)	0.0634 (3)	0.7865 (3)	0.0696 (10)	
O1B	0.5161 (2)	−0.3977 (2)	1.2444 (2)	0.0872 (9)	
O2B	0.5586 (3)	−0.5052 (2)	1.1192 (2)	0.0920 (10)	
O15B	0.7486 (3)	−0.3187 (3)	0.6696 (3)	0.1108 (12)	
S1B	0.57689 (9)	−0.43041 (8)	1.14932 (8)	0.0654 (3)	
S2B	0.72653 (8)	−0.23224 (7)	0.98706 (7)	0.0554 (3)	
Br1B	0.78611 (4)	−0.07405 (4)	1.05211 (4)	0.07916 (17)	
H8B	0.536 (3)	−0.278 (3)	1.095 (2)	0.043 (11)*	

### Atomic displacement parameters (Å^2^)

**Table:**

	*U*^11^	*U*^22^	*U*^33^	*U*^12^	*U*^13^	*U*^23^
C1A	0.078 (3)	0.050 (2)	0.061 (2)	−0.029 (2)	−0.036 (2)	−0.0098 (19)
C2A	0.119 (4)	0.061 (3)	0.065 (3)	−0.032 (3)	−0.028 (3)	−0.012 (2)
C3A	0.108 (4)	0.087 (4)	0.083 (4)	−0.016 (3)	−0.018 (3)	−0.035 (3)
C4A	0.108 (4)	0.116 (5)	0.071 (3)	−0.055 (4)	−0.014 (3)	−0.030 (3)
C5A	0.132 (5)	0.096 (4)	0.068 (3)	−0.073 (4)	−0.039 (3)	0.008 (3)
C6A	0.084 (3)	0.064 (3)	0.073 (3)	−0.035 (2)	−0.038 (3)	−0.002 (2)
C9A	0.061 (2)	0.048 (2)	0.053 (2)	−0.027 (2)	−0.0176 (19)	−0.0115 (17)
C10A	0.073 (3)	0.067 (3)	0.072 (3)	−0.038 (2)	−0.027 (2)	0.000 (2)
C11A	0.088 (3)	0.064 (3)	0.077 (3)	−0.040 (3)	−0.031 (3)	0.007 (2)
C12A	0.071 (3)	0.057 (3)	0.079 (3)	−0.015 (2)	−0.034 (2)	−0.012 (2)
O15A	0.091 (6)	0.085 (8)	0.151 (9)	−0.044 (4)	−0.048 (6)	−0.002 (6)
C16A	0.115 (8)	0.083 (6)	0.118 (7)	−0.033 (7)	−0.022 (7)	−0.014 (5)
O15C	0.057 (5)	0.091 (9)	0.143 (8)	−0.044 (5)	−0.046 (6)	0.009 (6)
C16C	0.127 (9)	0.087 (8)	0.138 (10)	−0.031 (7)	−0.088 (7)	0.052 (7)
C13A	0.062 (3)	0.066 (3)	0.071 (3)	−0.033 (2)	−0.018 (2)	−0.021 (2)
C14A	0.065 (3)	0.055 (2)	0.046 (2)	−0.029 (2)	−0.0159 (19)	−0.0131 (17)
C17A	0.0366 (19)	0.054 (2)	0.049 (2)	−0.0225 (17)	−0.0098 (16)	−0.0098 (17)
C19A	0.055 (2)	0.050 (2)	0.058 (2)	−0.0187 (19)	−0.0240 (19)	−0.0098 (18)
C21A	0.050 (2)	0.045 (2)	0.063 (2)	−0.0208 (18)	−0.0146 (19)	−0.0088 (19)
C22A	0.042 (2)	0.054 (2)	0.044 (2)	−0.0265 (18)	−0.0079 (16)	−0.0058 (17)
C24A	0.128 (4)	0.069 (3)	0.090 (3)	−0.023 (3)	−0.065 (3)	−0.024 (2)
C25A	0.131 (4)	0.109 (4)	0.092 (4)	−0.067 (4)	−0.059 (3)	0.002 (3)
C26A	0.139 (5)	0.092 (4)	0.128 (5)	−0.018 (4)	−0.089 (4)	−0.010 (4)
C27A	0.130 (5)	0.061 (3)	0.092 (4)	−0.010 (3)	−0.044 (3)	−0.001 (3)
C28A	0.139 (4)	0.062 (3)	0.082 (3)	−0.042 (3)	−0.056 (3)	−0.004 (2)
N8A	0.0604 (19)	0.0581 (19)	0.0576 (19)	−0.0255 (17)	−0.0184 (16)	−0.0138 (15)
N18A	0.0554 (18)	0.0496 (18)	0.0542 (18)	−0.0231 (15)	−0.0221 (15)	−0.0100 (15)
N20A	0.0557 (19)	0.0482 (19)	0.061 (2)	−0.0186 (16)	−0.0218 (16)	−0.0136 (15)
N23A	0.122 (3)	0.047 (2)	0.082 (2)	−0.021 (2)	−0.065 (2)	−0.0084 (17)
O1A	0.140 (3)	0.088 (2)	0.089 (2)	−0.082 (2)	−0.044 (2)	−0.0002 (17)
O2A	0.0668 (19)	0.102 (2)	0.106 (2)	−0.0290 (18)	−0.0446 (17)	−0.0161 (18)
S1A	0.0817 (8)	0.0697 (7)	0.0722 (7)	−0.0451 (6)	−0.0343 (6)	−0.0062 (5)
S2A	0.0795 (7)	0.0670 (6)	0.0474 (5)	−0.0445 (6)	−0.0170 (5)	−0.0086 (5)
Br2	0.0983 (3)	0.0802 (3)	0.0506 (2)	−0.0503 (3)	−0.0176 (2)	0.0050 (2)
C1B	0.072 (3)	0.049 (2)	0.071 (3)	−0.021 (2)	−0.034 (2)	−0.002 (2)
C2B	0.095 (4)	0.077 (3)	0.086 (3)	−0.028 (3)	−0.021 (3)	−0.023 (3)
C3B	0.080 (4)	0.096 (4)	0.116 (5)	−0.007 (3)	−0.006 (3)	−0.031 (3)
C4B	0.084 (4)	0.125 (6)	0.151 (6)	−0.025 (4)	−0.048 (4)	−0.008 (5)
C5B	0.100 (5)	0.136 (6)	0.190 (7)	−0.012 (4)	−0.085 (5)	−0.058 (5)
C6B	0.079 (4)	0.102 (4)	0.167 (6)	−0.004 (3)	−0.057 (4)	−0.063 (4)
C9B	0.052 (2)	0.0350 (19)	0.063 (2)	−0.0157 (18)	−0.0234 (19)	−0.0076 (17)
C10B	0.062 (3)	0.047 (2)	0.084 (3)	−0.024 (2)	−0.032 (2)	−0.010 (2)
C11B	0.077 (3)	0.060 (3)	0.084 (3)	−0.018 (2)	−0.039 (3)	−0.028 (2)
C12B	0.071 (3)	0.067 (3)	0.069 (3)	−0.019 (2)	−0.021 (2)	−0.024 (2)
C13B	0.057 (2)	0.059 (2)	0.071 (3)	−0.022 (2)	−0.015 (2)	−0.019 (2)
C14B	0.048 (2)	0.0373 (19)	0.068 (3)	−0.0122 (18)	−0.0253 (19)	−0.0123 (17)
C16B	0.149 (6)	0.198 (7)	0.106 (5)	−0.069 (5)	−0.017 (4)	−0.073 (5)
C17B	0.0405 (19)	0.045 (2)	0.047 (2)	−0.0164 (17)	−0.0120 (16)	−0.0099 (16)
C19B	0.051 (2)	0.054 (2)	0.052 (2)	−0.0200 (19)	−0.0189 (18)	−0.0117 (18)
C21B	0.053 (2)	0.053 (2)	0.066 (2)	−0.024 (2)	−0.0200 (19)	−0.0179 (19)
C22B	0.046 (2)	0.050 (2)	0.053 (2)	−0.0206 (18)	−0.0168 (17)	−0.0153 (17)
C24B	0.093 (3)	0.074 (3)	0.093 (3)	−0.037 (3)	−0.054 (3)	−0.013 (2)
C25B	0.078 (3)	0.153 (6)	0.130 (5)	−0.053 (4)	0.004 (4)	−0.080 (5)
C26B	0.183 (7)	0.218 (8)	0.060 (3)	−0.142 (6)	0.007 (4)	−0.038 (4)
C27B	0.129 (5)	0.151 (5)	0.088 (4)	−0.091 (4)	−0.024 (4)	0.008 (4)
C28B	0.080 (3)	0.072 (3)	0.075 (3)	−0.029 (2)	−0.039 (2)	0.000 (2)
N8B	0.063 (2)	0.044 (2)	0.069 (2)	−0.0237 (18)	−0.0241 (18)	−0.0068 (17)
N18B	0.0494 (17)	0.0500 (18)	0.0552 (18)	−0.0203 (15)	−0.0207 (15)	−0.0107 (14)
N20B	0.0531 (18)	0.0488 (18)	0.0597 (19)	−0.0193 (15)	−0.0220 (15)	−0.0133 (14)
N23B	0.089 (2)	0.057 (2)	0.085 (2)	−0.0271 (19)	−0.053 (2)	−0.0065 (18)
O1B	0.082 (2)	0.092 (2)	0.068 (2)	−0.0320 (18)	−0.0142 (16)	0.0052 (16)
O2B	0.121 (3)	0.0620 (18)	0.131 (3)	−0.0573 (19)	−0.072 (2)	0.0182 (17)
O15B	0.116 (3)	0.145 (3)	0.080 (2)	−0.040 (2)	−0.025 (2)	−0.053 (2)
S1B	0.0707 (7)	0.0570 (6)	0.0778 (8)	−0.0323 (6)	−0.0318 (6)	0.0054 (5)
S2B	0.0585 (6)	0.0522 (6)	0.0688 (6)	−0.0244 (5)	−0.0297 (5)	−0.0067 (5)
Br1B	0.0961 (3)	0.0770 (3)	0.0988 (4)	−0.0401 (3)	−0.0633 (3)	−0.0036 (2)

### Geometric parameters (Å, º)

**Table:**

C1A—C6A	1.364 (5)	O1A—S1A	1.427 (3)
C1A—C2A	1.383 (5)	O2A—S1A	1.435 (3)
C1A—S1A	1.753 (4)	C1B—C6B	1.358 (6)
C2A—C3A	1.380 (6)	C1B—C2B	1.366 (6)
C2A—H2A	0.9300	C1B—S1B	1.756 (4)
C3A—C4A	1.360 (7)	C2B—C3B	1.391 (7)
C3A—H3A	0.9300	C2B—H2B	0.9300
C4A—C5A	1.364 (6)	C3B—C4B	1.351 (8)
C4A—H4A	0.9300	C3B—H3B	0.9300
C5A—C6A	1.381 (6)	C4B—C5B	1.334 (8)
C5A—H5A	0.9300	C4B—H4B	0.9300
C6A—H6A	0.9300	C5B—C6B	1.387 (7)
C9A—C14A	1.386 (5)	C5B—H5B	0.9300
C9A—C10A	1.387 (5)	C6B—H6B	0.9300
C9A—N8A	1.429 (4)	C9B—C14B	1.382 (5)
C10A—C11A	1.360 (5)	C9B—C10B	1.393 (5)
C10A—H10A	0.9300	C9B—N8B	1.423 (5)
C11A—C12A	1.370 (6)	C10B—C11B	1.388 (5)
C11A—H11A	0.9300	C10B—H10B	0.9300
C12A—O15C	1.34 (2)	C11B—C12B	1.367 (6)
C12A—C13A	1.388 (5)	C11B—H11B	0.9300
C12A—O15A	1.404 (17)	C12B—C13B	1.375 (5)
O15A—C16A	1.311 (14)	C12B—O15B	1.385 (5)
C16A—H16D	0.9600	C13B—C14B	1.392 (5)
C16A—H16E	0.9600	C13B—H13B	0.9300
C16A—H16F	0.9600	C14B—S2B	1.774 (4)
O15C—C16C	1.421 (16)	C16B—O15B	1.358 (6)
C16C—H16G	0.9600	C16B—H16A	0.9600
C16C—H16H	0.9600	C16B—H16B	0.9600
C16C—H16I	0.9600	C16B—H16C	0.9600
C13A—C14A	1.385 (5)	C17B—N18B	1.315 (4)
C13A—H13A	0.9300	C17B—C22B	1.391 (5)
C14A—S2A	1.778 (4)	C17B—S2B	1.766 (3)
C17A—N18A	1.313 (4)	C19B—N20B	1.350 (4)
C17A—C22A	1.391 (5)	C19B—N18B	1.351 (4)
C17A—S2A	1.767 (4)	C19B—N23B	1.353 (4)
C19A—N23A	1.344 (4)	C21B—N20B	1.330 (4)
C19A—N18A	1.346 (4)	C21B—C22B	1.363 (5)
C19A—N20A	1.349 (4)	C21B—H21B	0.9300
C21A—N20A	1.324 (4)	C22B—Br1B	1.879 (3)
C21A—C22A	1.373 (5)	C24B—N23B	1.464 (5)
C21A—H21A	0.9300	C24B—C25B	1.478 (6)
C22A—Br2	1.879 (3)	C24B—H24A	0.9700
C24A—C25A	1.440 (6)	C24B—H24B	0.9700
C24A—N23A	1.451 (5)	C25B—C26B	1.501 (8)
C24A—H24C	0.9700	C25B—H25C	0.9700
C24A—H24D	0.9700	C25B—H25D	0.9700
C25A—C26A	1.490 (7)	C26B—C27B	1.526 (7)
C25A—H25A	0.9700	C26B—H26A	0.9700
C25A—H25B	0.9700	C26B—H26B	0.9700
C26A—C27A	1.511 (7)	C27B—C28B	1.498 (6)
C26A—H26C	0.9700	C27B—H27C	0.9700
C26A—H26D	0.9700	C27B—H27D	0.9700
C27A—C28A	1.485 (6)	C28B—N23B	1.448 (5)
C27A—H27A	0.9700	C28B—H28A	0.9700
C27A—H27B	0.9700	C28B—H28B	0.9700
C28A—N23A	1.461 (5)	N8B—S1B	1.622 (3)
C28A—H28C	0.9700	N8B—H8B	0.78 (3)
C28A—H28D	0.9700	O1B—S1B	1.430 (3)
N8A—S1A	1.621 (3)	O2B—S1B	1.426 (3)
N8A—H8A	0.8600		
			
C6A—C1A—C2A	119.3 (4)	O2A—S1A—N8A	105.92 (17)
C6A—C1A—S1A	120.1 (3)	O1A—S1A—C1A	106.80 (19)
C2A—C1A—S1A	120.5 (3)	O2A—S1A—C1A	108.87 (19)
C3A—C2A—C1A	119.9 (4)	N8A—S1A—C1A	105.93 (16)
C3A—C2A—H2A	120.1	C17A—S2A—C14A	100.74 (16)
C1A—C2A—H2A	120.1	C6B—C1B—C2B	119.3 (4)
C4A—C3A—C2A	120.3 (5)	C6B—C1B—S1B	120.4 (4)
C4A—C3A—H3A	119.8	C2B—C1B—S1B	120.4 (4)
C2A—C3A—H3A	119.8	C1B—C2B—C3B	119.5 (5)
C3A—C4A—C5A	119.9 (5)	C1B—C2B—H2B	120.3
C3A—C4A—H4A	120.1	C3B—C2B—H2B	120.3
C5A—C4A—H4A	120.1	C4B—C3B—C2B	119.8 (5)
C4A—C5A—C6A	120.3 (5)	C4B—C3B—H3B	120.1
C4A—C5A—H5A	119.8	C2B—C3B—H3B	120.1
C6A—C5A—H5A	119.8	C5B—C4B—C3B	121.3 (6)
C1A—C6A—C5A	120.2 (4)	C5B—C4B—H4B	119.4
C1A—C6A—H6A	119.9	C3B—C4B—H4B	119.4
C5A—C6A—H6A	119.9	C4B—C5B—C6B	119.3 (6)
C14A—C9A—C10A	118.9 (4)	C4B—C5B—H5B	120.4
C14A—C9A—N8A	122.5 (3)	C6B—C5B—H5B	120.4
C10A—C9A—N8A	118.6 (4)	C1B—C6B—C5B	120.8 (5)
C11A—C10A—C9A	121.3 (4)	C1B—C6B—H6B	119.6
C11A—C10A—H10A	119.3	C5B—C6B—H6B	119.6
C9A—C10A—H10A	119.3	C14B—C9B—C10B	118.7 (4)
C10A—C11A—C12A	119.7 (4)	C14B—C9B—N8B	122.2 (3)
C10A—C11A—H11A	120.1	C10B—C9B—N8B	119.1 (3)
C12A—C11A—H11A	120.1	C11B—C10B—C9B	120.4 (4)
O15C—C12A—C11A	129.2 (7)	C11B—C10B—H10B	119.8
O15C—C12A—C13A	110.2 (7)	C9B—C10B—H10B	119.8
C11A—C12A—C13A	120.5 (4)	C12B—C11B—C10B	120.1 (4)
O15C—C12A—O15A	20.6 (9)	C12B—C11B—H11B	119.9
C11A—C12A—O15A	108.8 (6)	C10B—C11B—H11B	119.9
C13A—C12A—O15A	130.6 (6)	C11B—C12B—C13B	120.3 (4)
C16A—O15A—C12A	110.2 (11)	C11B—C12B—O15B	125.1 (4)
O15A—C16A—H16D	109.5	C13B—C12B—O15B	114.6 (4)
O15A—C16A—H16E	109.5	C12B—C13B—C14B	119.9 (4)
H16D—C16A—H16E	109.5	C12B—C13B—H13B	120.1
O15A—C16A—H16F	109.5	C14B—C13B—H13B	120.1
H16D—C16A—H16F	109.5	C9B—C14B—C13B	120.5 (3)
H16E—C16A—H16F	109.5	C9B—C14B—S2B	120.8 (3)
C12A—O15C—C16C	116.7 (13)	C13B—C14B—S2B	118.7 (3)
O15C—C16C—H16G	109.5	O15B—C16B—H16A	109.5
O15C—C16C—H16H	109.5	O15B—C16B—H16B	109.5
H16G—C16C—H16H	109.5	H16A—C16B—H16B	109.5
O15C—C16C—H16I	109.5	O15B—C16B—H16C	109.5
H16G—C16C—H16I	109.5	H16A—C16B—H16C	109.5
H16H—C16C—H16I	109.5	H16B—C16B—H16C	109.5
C14A—C13A—C12A	119.4 (4)	N18B—C17B—C22B	121.9 (3)
C14A—C13A—H13A	120.3	N18B—C17B—S2B	119.1 (3)
C12A—C13A—H13A	120.3	C22B—C17B—S2B	119.0 (3)
C13A—C14A—C9A	120.0 (3)	N20B—C19B—N18B	125.0 (3)
C13A—C14A—S2A	119.9 (3)	N20B—C19B—N23B	118.3 (3)
C9A—C14A—S2A	120.1 (3)	N18B—C19B—N23B	116.7 (3)
N18A—C17A—C22A	121.1 (3)	N20B—C21B—C22B	124.1 (4)
N18A—C17A—S2A	119.8 (3)	N20B—C21B—H21B	118.0
C22A—C17A—S2A	119.2 (3)	C22B—C21B—H21B	118.0
N23A—C19A—N18A	116.9 (3)	C21B—C22B—C17B	116.4 (3)
N23A—C19A—N20A	117.9 (3)	C21B—C22B—Br1B	122.5 (3)
N18A—C19A—N20A	125.1 (3)	C17B—C22B—Br1B	121.0 (3)
N20A—C21A—C22A	123.9 (3)	N23B—C24B—C25B	110.5 (4)
N20A—C21A—H21A	118.0	N23B—C24B—H24A	109.6
C22A—C21A—H21A	118.0	C25B—C24B—H24A	109.6
C21A—C22A—C17A	116.7 (3)	N23B—C24B—H24B	109.6
C21A—C22A—Br2	120.7 (3)	C25B—C24B—H24B	109.6
C17A—C22A—Br2	122.6 (3)	H24A—C24B—H24B	108.1
C25A—C24A—N23A	112.9 (4)	C24B—C25B—C26B	111.2 (4)
C25A—C24A—H24C	109.0	C24B—C25B—H25C	109.4
N23A—C24A—H24C	109.0	C26B—C25B—H25C	109.4
C25A—C24A—H24D	109.0	C24B—C25B—H25D	109.4
N23A—C24A—H24D	109.0	C26B—C25B—H25D	109.4
H24C—C24A—H24D	107.8	H25C—C25B—H25D	108.0
C24A—C25A—C26A	112.6 (5)	C25B—C26B—C27B	110.3 (5)
C24A—C25A—H25A	109.1	C25B—C26B—H26A	109.6
C26A—C25A—H25A	109.1	C27B—C26B—H26A	109.6
C24A—C25A—H25B	109.1	C25B—C26B—H26B	109.6
C26A—C25A—H25B	109.1	C27B—C26B—H26B	109.6
H25A—C25A—H25B	107.8	H26A—C26B—H26B	108.1
C25A—C26A—C27A	110.5 (4)	C28B—C27B—C26B	111.6 (4)
C25A—C26A—H26C	109.6	C28B—C27B—H27C	109.3
C27A—C26A—H26C	109.6	C26B—C27B—H27C	109.3
C25A—C26A—H26D	109.6	C28B—C27B—H27D	109.3
C27A—C26A—H26D	109.6	C26B—C27B—H27D	109.3
H26C—C26A—H26D	108.1	H27C—C27B—H27D	108.0
C28A—C27A—C26A	110.9 (4)	N23B—C28B—C27B	109.9 (4)
C28A—C27A—H27A	109.5	N23B—C28B—H28A	109.7
C26A—C27A—H27A	109.5	C27B—C28B—H28A	109.7
C28A—C27A—H27B	109.5	N23B—C28B—H28B	109.7
C26A—C27A—H27B	109.5	C27B—C28B—H28B	109.7
H27A—C27A—H27B	108.0	H28A—C28B—H28B	108.2
N23A—C28A—C27A	109.9 (4)	C9B—N8B—S1B	121.4 (3)
N23A—C28A—H28C	109.7	C9B—N8B—H8B	116 (3)
C27A—C28A—H28C	109.7	S1B—N8B—H8B	111 (3)
N23A—C28A—H28D	109.7	C17B—N18B—C19B	117.3 (3)
C27A—C28A—H28D	109.7	C21B—N20B—C19B	115.2 (3)
H28C—C28A—H28D	108.2	C19B—N23B—C28B	122.7 (4)
C9A—N8A—S1A	120.2 (2)	C19B—N23B—C24B	121.5 (3)
C9A—N8A—H8A	119.9	C28B—N23B—C24B	113.7 (3)
S1A—N8A—H8A	119.9	C16B—O15B—C12B	118.7 (5)
C17A—N18A—C19A	118.0 (3)	O2B—S1B—O1B	120.6 (2)
C21A—N20A—C19A	115.1 (3)	O2B—S1B—N8B	107.80 (19)
C19A—N23A—C24A	123.3 (3)	O1B—S1B—N8B	105.81 (19)
C19A—N23A—C28A	122.0 (3)	O2B—S1B—C1B	106.8 (2)
C24A—N23A—C28A	114.7 (3)	O1B—S1B—C1B	107.7 (2)
O1A—S1A—O2A	120.56 (19)	N8B—S1B—C1B	107.61 (18)
O1A—S1A—N8A	107.91 (17)	C17B—S2B—C14B	100.68 (16)
			
C6A—C1A—C2A—C3A	−1.1 (6)	C22A—C17A—S2A—C14A	168.4 (3)
S1A—C1A—C2A—C3A	175.5 (4)	C13A—C14A—S2A—C17A	72.9 (3)
C1A—C2A—C3A—C4A	1.1 (8)	C9A—C14A—S2A—C17A	−107.4 (3)
C2A—C3A—C4A—C5A	−0.2 (8)	C6B—C1B—C2B—C3B	2.0 (7)
C3A—C4A—C5A—C6A	−0.6 (8)	S1B—C1B—C2B—C3B	−178.3 (4)
C2A—C1A—C6A—C5A	0.2 (6)	C1B—C2B—C3B—C4B	−1.0 (9)
S1A—C1A—C6A—C5A	−176.4 (3)	C2B—C3B—C4B—C5B	−0.3 (11)
C4A—C5A—C6A—C1A	0.6 (7)	C3B—C4B—C5B—C6B	0.5 (11)
C14A—C9A—C10A—C11A	0.4 (6)	C2B—C1B—C6B—C5B	−1.8 (9)
N8A—C9A—C10A—C11A	−179.1 (4)	S1B—C1B—C6B—C5B	178.4 (5)
C9A—C10A—C11A—C12A	1.4 (6)	C4B—C5B—C6B—C1B	0.6 (11)
C10A—C11A—C12A—O15C	−178.5 (17)	C14B—C9B—C10B—C11B	−1.3 (5)
C10A—C11A—C12A—C13A	−1.2 (7)	N8B—C9B—C10B—C11B	178.3 (3)
C10A—C11A—C12A—O15A	177.8 (11)	C9B—C10B—C11B—C12B	−0.8 (6)
O15C—C12A—O15A—C16A	4 (5)	C10B—C11B—C12B—C13B	2.5 (6)
C11A—C12A—O15A—C16A	176.4 (14)	C10B—C11B—C12B—O15B	−177.5 (4)
C13A—C12A—O15A—C16A	−5 (3)	C11B—C12B—C13B—C14B	−2.0 (6)
C11A—C12A—O15C—C16C	2 (3)	O15B—C12B—C13B—C14B	178.0 (3)
C13A—C12A—O15C—C16C	−175.4 (18)	C10B—C9B—C14B—C13B	1.8 (5)
O15A—C12A—O15C—C16C	12 (4)	N8B—C9B—C14B—C13B	−177.8 (3)
O15C—C12A—C13A—C14A	177.0 (14)	C10B—C9B—C14B—S2B	−178.7 (3)
C11A—C12A—C13A—C14A	−0.8 (6)	N8B—C9B—C14B—S2B	1.7 (5)
O15A—C12A—C13A—C14A	−179.6 (13)	C12B—C13B—C14B—C9B	−0.1 (5)
C12A—C13A—C14A—C9A	2.6 (5)	C12B—C13B—C14B—S2B	−179.6 (3)
C12A—C13A—C14A—S2A	−177.8 (3)	N20B—C21B—C22B—C17B	−1.8 (5)
C10A—C9A—C14A—C13A	−2.4 (5)	N20B—C21B—C22B—Br1B	176.9 (3)
N8A—C9A—C14A—C13A	177.1 (3)	N18B—C17B—C22B—C21B	1.3 (5)
C10A—C9A—C14A—S2A	178.0 (3)	S2B—C17B—C22B—C21B	−179.4 (3)
N8A—C9A—C14A—S2A	−2.5 (5)	N18B—C17B—C22B—Br1B	−177.4 (3)
N20A—C21A—C22A—C17A	−2.7 (5)	S2B—C17B—C22B—Br1B	1.9 (4)
N20A—C21A—C22A—Br2	179.0 (3)	N23B—C24B—C25B—C26B	−56.1 (6)
N18A—C17A—C22A—C21A	3.0 (5)	C24B—C25B—C26B—C27B	54.2 (7)
S2A—C17A—C22A—C21A	−177.3 (2)	C25B—C26B—C27B—C28B	−53.4 (7)
N18A—C17A—C22A—Br2	−178.7 (2)	C26B—C27B—C28B—N23B	54.0 (6)
S2A—C17A—C22A—Br2	1.0 (4)	C14B—C9B—N8B—S1B	−107.0 (4)
N23A—C24A—C25A—C26A	51.0 (6)	C10B—C9B—N8B—S1B	73.5 (4)
C24A—C25A—C26A—C27A	−53.1 (7)	C22B—C17B—N18B—C19B	1.9 (5)
C25A—C26A—C27A—C28A	55.4 (7)	S2B—C17B—N18B—C19B	−177.5 (2)
C26A—C27A—C28A—N23A	−55.0 (6)	N20B—C19B—N18B—C17B	−5.0 (5)
C14A—C9A—N8A—S1A	−98.8 (4)	N23B—C19B—N18B—C17B	177.5 (3)
C10A—C9A—N8A—S1A	80.7 (4)	C22B—C21B—N20B—C19B	−0.9 (5)
C22A—C17A—N18A—C19A	−0.2 (5)	N18B—C19B—N20B—C21B	4.5 (5)
S2A—C17A—N18A—C19A	−179.9 (3)	N23B—C19B—N20B—C21B	−178.0 (3)
N23A—C19A—N18A—C17A	176.7 (3)	N20B—C19B—N23B—C28B	12.8 (6)
N20A—C19A—N18A—C17A	−3.2 (5)	N18B—C19B—N23B—C28B	−169.5 (3)
C22A—C21A—N20A—C19A	−0.3 (5)	N20B—C19B—N23B—C24B	175.6 (3)
N23A—C19A—N20A—C21A	−176.4 (3)	N18B—C19B—N23B—C24B	−6.7 (6)
N18A—C19A—N20A—C21A	3.5 (5)	C27B—C28B—N23B—C19B	106.9 (5)
N18A—C19A—N23A—C24A	176.7 (4)	C27B—C28B—N23B—C24B	−57.1 (5)
N20A—C19A—N23A—C24A	−3.3 (6)	C25B—C24B—N23B—C19B	−105.7 (5)
N18A—C19A—N23A—C28A	−3.6 (6)	C25B—C24B—N23B—C28B	58.5 (5)
N20A—C19A—N23A—C28A	176.3 (4)	C11B—C12B—O15B—C16B	14.2 (7)
C25A—C24A—N23A—C19A	127.6 (5)	C13B—C12B—O15B—C16B	−165.9 (5)
C25A—C24A—N23A—C28A	−52.1 (6)	C9B—N8B—S1B—O2B	−55.9 (4)
C27A—C28A—N23A—C19A	−126.0 (4)	C9B—N8B—S1B—O1B	173.8 (3)
C27A—C28A—N23A—C24A	53.7 (6)	C9B—N8B—S1B—C1B	58.9 (4)
C9A—N8A—S1A—O1A	−53.6 (3)	C6B—C1B—S1B—O2B	−160.6 (4)
C9A—N8A—S1A—O2A	176.0 (3)	C2B—C1B—S1B—O2B	19.7 (4)
C9A—N8A—S1A—C1A	60.5 (3)	C6B—C1B—S1B—O1B	−29.7 (5)
C6A—C1A—S1A—O1A	−151.9 (3)	C2B—C1B—S1B—O1B	150.5 (3)
C2A—C1A—S1A—O1A	31.5 (4)	C6B—C1B—S1B—N8B	83.9 (4)
C6A—C1A—S1A—O2A	−20.3 (4)	C2B—C1B—S1B—N8B	−95.9 (4)
C2A—C1A—S1A—O2A	163.2 (3)	N18B—C17B—S2B—C14B	4.3 (3)
C6A—C1A—S1A—N8A	93.2 (3)	C22B—C17B—S2B—C14B	−175.1 (3)
C2A—C1A—S1A—N8A	−83.3 (3)	C9B—C14B—S2B—C17B	−109.7 (3)
N18A—C17A—S2A—C14A	−11.8 (3)	C13B—C14B—S2B—C17B	69.8 (3)

### Hydrogen-bond geometry (Å, º)

**Table:**

*D*—H···*A*	*D*—H	H···*A*	*D*···*A*	*D*—H···*A*
N8*A*—H8*A*···N20*A*^i^	0.86	2.15	2.940 (4)	152
N8*B*—H8*B*···N20*B*^ii^	0.78 (4)	2.28 (4)	2.974 (5)	149 (4)
C11*A*—H11*A*···O1*A*^iii^	0.93	2.47	3.372 (5)	164
C10*B*—H10*B*···O2*B*^iv^	0.93	2.60	3.278 (6)	131

Symmetry codes: (i) −*x*+2, −*y*, −*z*+1; (ii) −*x*+1, −*y*, −*z*+2; (iii) −*x*+2, −*y*+1, −*z*+1; (iv) −*x*+1, −*y*−1, −*z*+2.
